# Development of ISSR-derived SCAR Marker and SYBR Green I Real-time PCR Method for Detection of Teliospores of *Tilletia laevis* Kühn

**DOI:** 10.1038/s41598-019-54163-5

**Published:** 2019-11-27

**Authors:** Zhaoqun Yao, Dandan Qin, Delai Chen, Changzhong Liu, Wanquan Chen, Taiguo Liu, Bo Liu, Li Gao

**Affiliations:** 1 State Key Laboratory for Biology of Plant Disease and Insect Pests, Institute of Plant Protection, Chinese Academy of Agricultural Sciences, 100193 Beijing, China; 2 Key Laboratory at Universities of Xinjiang Uygur Autonomous Region for Oasis Agricultural Pest Management and Plant Protection Resource Utilization, Shihezi University, 832003 Shihezi, China; 3 College of Plant Protection, Gansu Agricultural University, 730070 Lanzhou, China

**Keywords:** Fungi, Pathogens

## Abstract

Common bunt, caused by *Tilletia laevis* Kühn [syn. *T*. *foetida* (Wallr) Liro] and *Tilletia tritici* (Bjerk.) Wint. [syn. *T*. *caries* (DC) Tul.], is an important wheat disease worldwide. To quickly differentiate the closely related fungi *T*. *laevis*, *T*. *tritici* and *Tilletia controversa* (a pathogen that causes dwarf bunt of wheat and has been requested as a quarantined pathogen in many countries), a rapid diagnostic and detection method for an ISSR molecular marker was developed for the first time in this study. Based on the *T*. *laevis*-specific band (1300 bp) amplified by the primer ISSR860, a pair of SCAR primers (L60F/L60R) was designed to amplify a specific 660-bp DNA fragment from the isolates of *T*. *laevis* but not other related pathogens. The detection limit of the SCAR marker was 0.4 ng/μl of DNA from *T*. *laevis*; moreover, a SYBR Green I real-time PCR method was also successfully developed based on the SCAR marker with the detection limit of 10 fg/μl *T*. *laevis* DNA. This is the first report of a rapid, specific and highly sensitive SCAR marker and SYBR Green I real-time PCR method for detection of the teliospores of *T*. *laevis* based on ISSR technology. This method allows highly efficient, rapid and accurate differentiation of the pathogen from related pathogens, especially from the very similar pathogens *T*. *tritici* and *T*. *controversa*.

## Introduction

Contamination of wheat with common bunt spores has resulted in considerable loss of yield and seed quality accompanied by a fish-like odor^1,2^. Typically, yield loss is proportional to disease incidence because wheat kernels are replaced by bunt spores. If untreated seeds are used, the incidence of common bunt can reach 70% to 80%, with yield loss of 41% observed in Romania^3^, and yield losses reaching 10–20% and 25–30% in Turkey and Iran, respectively^4^.

Common bunt is caused by the pathogens *Tilletia laevis* Kühn [syn. *T*. *foetida* (Wallr) Liro] and *Tilletia tritici* (Bjerk.) Wint. [syn. *T*. *caries* (DC) Tul.], both could be variants of the same species together with the pathogen that causes dwarf bunt, *Tilletia controversa* Kühn, as proposed by several genetic, biochemical, and molecular studies^5^. In particular, *T*. *laevis*, and *T*. *tritici*, are very similar in terms of germination requirements, lifestyles, and disease symptoms^2^. Many traditional methods for pathogen detection have mostly been used for analysis of morphological and physiological features, such as microscopic examination of teliospores^6^, immunological detection methods^7^, and analyses of the polypeptide profiles^8^, triacylglycerol profiles^9^ and genetic properties of teliospores^10^. These characteristics are usually used as taxonomic criteria to diagnose and detect pathogens^11^, but these methods are time consuming and exhibit low diagnostic accuracy for the detection of *T*. *laevis*. For example, based on microscopic observation, the cell wall surface of the teliospore of *T*. *laevis* was smooth, whereas that of *T*. *caries* was reticular. The mean diameter of *T*. *caries* teliospores is 14–23.5 μm, occasionally 25 μm. Similarly, the diameter of *T*. *laevis* teliospores ranges from 13 to 22 μm^12^. *T*. *controversa* has a similar morphology and genetic structure to those of *T*. *caries*^13^. The development of an efficient method for rapid and accurate pathogen detection is urgently needed.

Detection technologies, such as molecular, enzymatic, chromatographic and spectroscopic detection, are more sensitive, precise and time saving than traditional detection methods. In recent studies, researchers tried to distinguish *T*. *tritici*, *T*. *laevis*, *T*. *controversa*, and *Tilletia bromi* with species-specific primers in the ITS region, in the large subunit of the ribosomal RNA genes and RPB2 genes, but they failed because no variation was observed among the regions^14,15^. However, Kochanová *et al*.^16^ developed PCR primers for the ITS1 region of *Tilletia* species for simultaneous detection of *T*. *controversa* and *T*. *tritici*. Vesna *et al*.^17^ reported the differentiation of *Tilletia* species by repetitive sequence-based polymerase chain reaction (rep-PCR) fingerprinting. However, many molecular markers have been developed to differentiate *T*. *controversa* from similar species, including *T*. *laevis* and *T*. *controversa*, based on AFLP, RAPD, and ISSR analyses^11,13,18–20^. To date, there has been no report on molecular markers that could distinguish *T*. *laevis* from similar species of pathogens obtained by ISSR analysis, such as *T*. *tritici* and *T*. *controversa*.

Therefore, the main aim and objectives of this study were to develop a detection method that could be rapid, reliable and sensitive for distinguishing the teliospores of *T*. *laevis* from those of similar species (especially *T*. *tritici* and *T*. *controversa*) by ISSR technology, which will contribute to a diagnostic method based on a SCAR marker and SYBR Green I real-time PCR.

## Results

### Development of the SCAR marker

**Figure 1:**
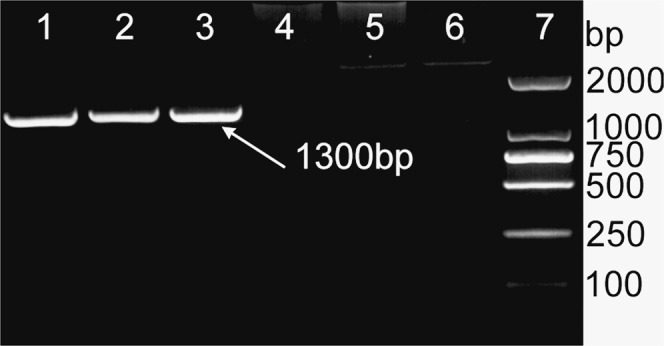
ISSR analysis was performed with DNA from *Tilletia laevis*, *Tilletia controversa* and *Tilletia caries*. The ISSR patterns of *Tilletia laevis*, *Tilletia controversa* and *Tilletia caries* were amplified by the ISSR860 primer. Lanes 1–3: *Tilletia laevis*, lane 4: ddH_2_O, lane 5: *Tilletia controversa*, lane 6: *Tilletia tritici*, lane 7: DL2000 DNA ladder.

**Figure 2:**
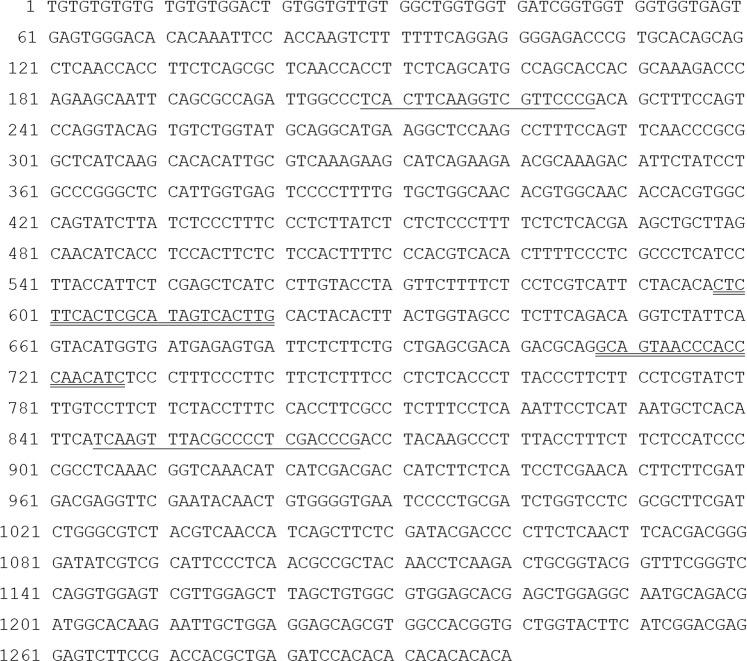
Sequence of the specific DNA fragment of *Tilletia laevis*. Underlined were the designed sequence-characterized amplified region (SCAR) primers (L60F and L60R). Underlined with double lines were RT-PCR primers qL60F/qL60R.

In this study, *T*. *laevis* was differentiated from *T*. *controversa* by a polymorphic profile (1300 bp), which was produced by the ISSR 860 primer (Fig. 1). Based on the specific DNA sequence of *T*. *laevis* (Fig. 2), the SCAR primer pair was designed by Primer-BLAST and named L60F (5′-TCACTTCAAGGTCGTTCCCG-3′)/L60R (5′-GTCGAGGGGCGTAAACTTGA-3′).

### Specificity and detection limit of the SCAR marker

**Figure 3:**
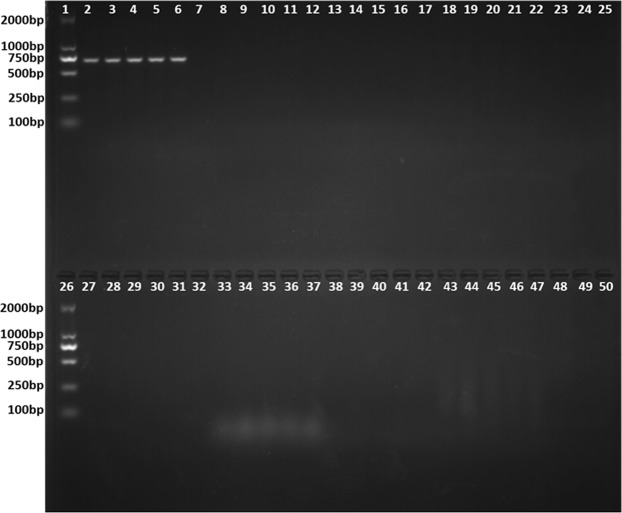
Specificity of the SCAR marker. Lane 1, 26-D2000 DNA Marker, lanes 2–6: *Tilletia laevis*, lane 7–11: *Tilletia controversa*, lanes 12–15: *Tilletia tritici*; lanes 16–18: *Puccinia striiformis* f. sp. *tritici*, lanes 19–21: *Puccinia triticina*, lanes 22–25 and 27: *Ustilago maydis*, lanes 28–32: *Ustilago horde*, lanes 33–37: *Blumeria graminis*, lanes 38–42: *Fusarium graminearum*, lanes 43–47: *Ustilago nuda*, lanes 48–50: ddH_2_O.

**Figure 4:**
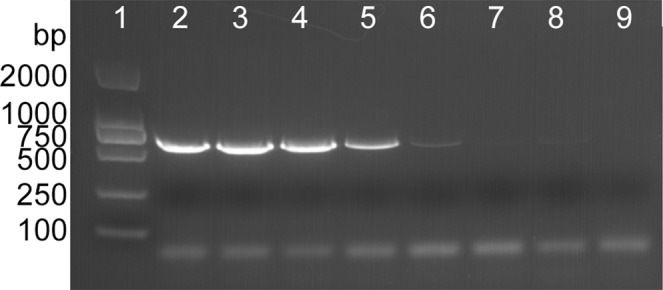
Sensitivity of the SCAR marker (L60F/L60R) with different amounts of DNA template. Lane 1: DL2000 DNA ladder, lane 2: 4 ng/μl, lane 3: 2 ng/μl, lane 4: 1 ng/μl, lane 5: 0.4 ng/μl, lane 6: 0.04 ng/μl, lane 7: 4 pg/μl, lane 8: 0.4 pg/μl, lane 9: ddH_2_O.

The SCAR primer amplified a specific 660-bp band from *T*. *laevis* but no products from other related species (*T*. *tritici*, *T*. *controversa*, *U*. *maydis*, *U*. *horde*, *U*. *nuda*, *P*. *striiformis* f. sp. *tritici*, *P*. *triticina*, *B*. *graminis* and *F*. *graminearum*) (Fig. 3), and the detection limit of the primer was 0.4 ng/μl in the PCR mixture using genomic DNA extracted from *T*. *laevis* (Fig. 4).

### Development of the SYBR Green I real-time PCR detection method

**Figure 5:**
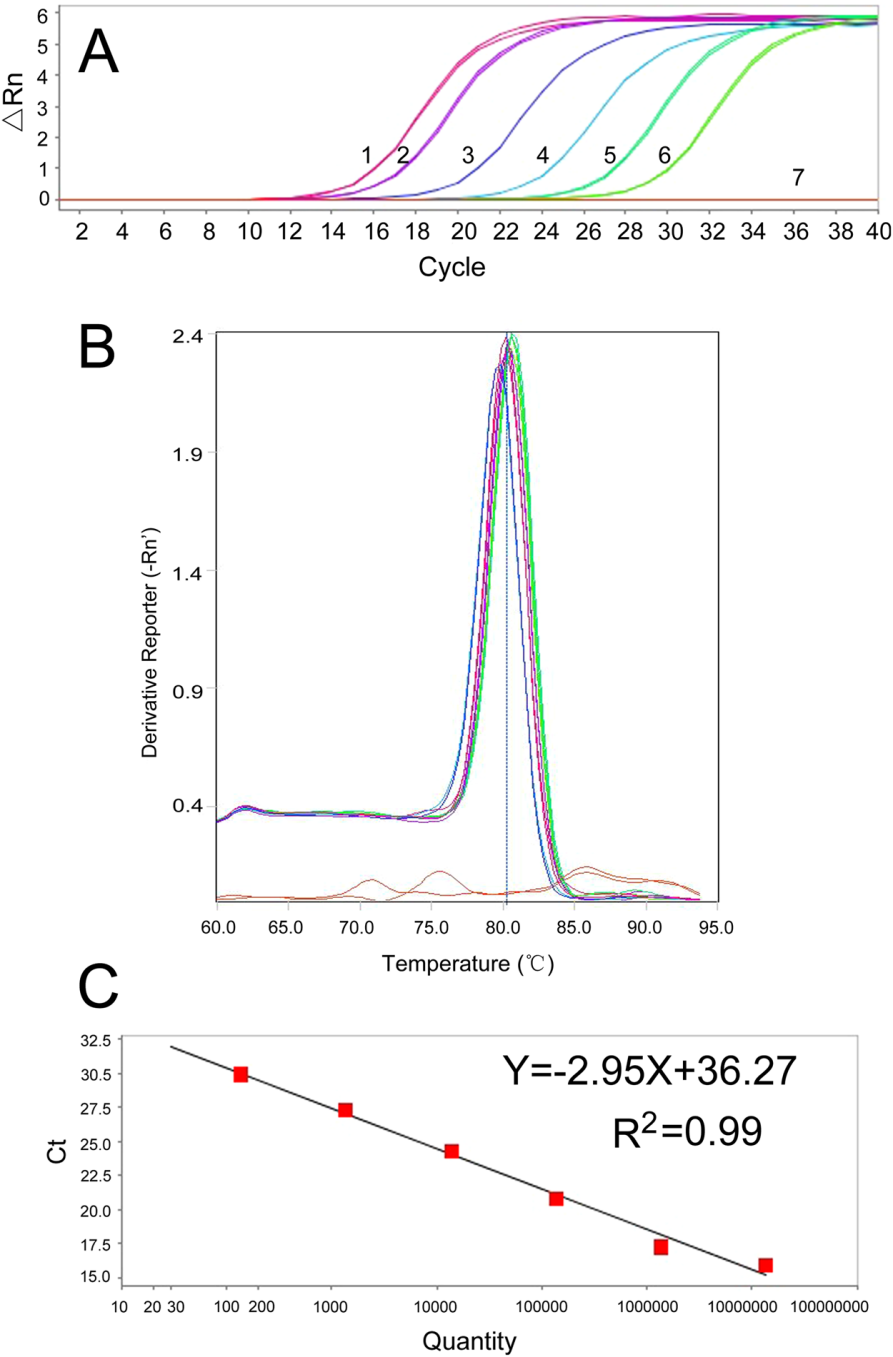
Establishment of the standard curve by SYBR Green I RT-PCR. (**A**) Real-time amplification curves. 1–6: ten-fold dilutions of recombined plasmid DNA (1.37 × 10^7^–1.37 × 10^2^ copies/μl; 0.01 ng–0.1 fg); 7: negative control. (**B**) Melt curve of SYBR Green I (peak temperature at 80 °C). (**C**) Standard curve.

Based on the SCAR marker, the SYBR Green I real-time PCR method for identification of *T*. *laevis* teliospore DNA was successfully developed in this study. The standard curve was generated with a linear range covering 6 log units (Fig. 5A). The melt curve of SYBR Green I is shown in Fig. 5B. The correlation coefficient of the standard curve in SYBR Green I RT-PCR reached 0.99 (Fig. 5C). Moreover, the amplification was specific, as shown by the melt curve, and the detection sensitivity reached 10 fg/μl (1.37 × 10^2^ copies/μl). These results demonstrated that a sensitive real-time PCR detection method for *T*. *laevis* teliospores was successfully established with SYBR Green I.

## Discussion

In this study, a species-specific DNA region (1300 bp) of *T*. *laevis* was successfully discovered using the ISSR 860 primer, and a specific 660-bp SCAR marker of *T*. *laevis* was developed that could successfully differentiate *T*. *laevis* from 9 related fungi (40 isolates) in a PCR assay, namely, *U*. *maydis*, *U*. *horde*, *U*. *nuda*, *P*. *striiformis* f. sp. *tritici*, *P*. *triticina*, *B*. *graminis* and *F*. *graminearum*, and, in particular, the very similar species *T*. *tritici* and *T*. *controversa*. The detection limit of the SCAR primer set was 0.4 ng/μl. This sensitivity is almost 10 times higher than that of the reported SCAR marker (SC_286-1_/SC_286-2_, Fig. S1), which had a sensitivity of 4 ng/μl^21^ and was developed by AFLP technology. Based on the SCAR marker in this study, we also developed the SYBR Green I real-time PCR detection method with a sensitivity of 10 fg/μl, which is more sensitive than the PCR assay (Table S1).

Methods for identification and monitoring of pathogens must be rapid, simple and sensitive^22^. A variety of strategies have been employed to identify pathogens and diagnose diseases by using DNA molecular marker technologies, which can distinguish sequence variations in specific genomic regions. This method provides a powerful tool for identification of pathogens with high efficiency^23^. Although the ITS region of the ribosomal subunit has been used as a target for species-specific primers^24^, in the case of wheat bunt, it was not possible to design species-specific primers in the sequenced ITS region, as there is no variation between the ITS2 regions of *T*. *tritici*, *T*. *laevis*, *T*. *controversa*^15^. Compared with several other types of DNA marker technologies, e.g., RFLP, AFLP, RAPD and SSR, ISSR analysis exhibits relatively high consistency and repeatability^25^, which has already been successfully used to differentiate the very similar species *T*. *controversa*^13,19^ from *T*. *tritici* and *T*. *laevis*. However, to date, there have been no reports on the SCAR marker of *T*. *laevis* using ISSR technology, and this study revealed the possibility of identifying *T*. *laevis* using this method. In this study, a rapid, simple and accurate detection method was developed to distinguish *T*. *laevis* from *T*. *controversa* and *T*. *tritici* with a SCAR marker.

In summary, this is the first report of a rapid, specific and highly sensitive SCAR marker for detection of the teliospores of *T*. *laevis* from 9 related fungi (40 related isolates) by ISSR analysis, which will provide a highly efficient method for differentiation of the pathogen from very similar pathogens, including *T*. *tritici* and *T*. *controversa*. In the near future, we will focus on the development of a high-throughput kit for rapid, sensitive, and accurate detection of *T*. *laevis* from *T*. *tritici* and *T*. *controversa*, which will contribute significantly to for the great potential in the on-site detection.

## Materials and Methods

### Isolates and DNA extraction

The pathogen *T*. *laevis*; the very similar species *T*. *controversa* and *T*. *tritici*; the smut fungi *Ustilago maydis*, *Ustilago horde*, and *Ustilago nuda*; and fungal pathogens of wheat, including *Puccinia striiformis* f. sp. *tritici*, *Puccinia triticina*, *and Blumeria graminis*, in addition to *Fusarium graminearum*, were used in the study. Five isolates of *T*. *controversa* were collected in the United States by Blair Goates (National Small Grains Germplasm Research Facility, USDA-ARS). All other fungi were isolated by Prof. Li Gao in IPP, CAAS (Institute of Plant Protection, Chinese Academy of Agricultural Sciences). Genomic DNA was extracted according to Gao *et al*.^12^, with slight modifications (the teliospores were crushed with FastPrep-24 (MP, USA)). The final genomic DNA solution was treated with RNase and analyzed on an agarose gel^22^.

### ISSR procedure

The UBC primer set # 9 (100 primers; University of British Columbia) was tested for ISSR analysis. PCR amplification was conducted using a 25-μl reaction mixture containing 400 pmol of primer, 100 ng of template DNA, and 12.5 μl of 2 × EasyTaq PCR SuperMix (+dye) (TransGen Biotech, China) on a Gradient Thermocycler (Bio-Rad, USA) with the following program: an initial denaturing cycle (3 min at 94 °C), followed by 35 cycles of 30 s at 94 °C, 30 s at the annealing temperature (32–75 °C depending on the primer), and 2 min of elongation at 72 °C and a final extension (10 min at 72 °C). The amplification products were analyzed on 1.5% agarose gels at 180 V for 20 min in 0.5 × TBE buffer (20 mM/l Tris-boric acid, 1 mM/l EDTA, pH 8.0) containing 0.1 mg/ml ethidium bromide. Banding patterns were visualized under UV light and photographed using a gel imager (Bio-Rad, USA).

### Development of the SCAR marker

The specific band (1300 bp) generated of the *T*. *laevis* DNA generated by the primer ISSR860 (5′-TGTGTGTGTGTGTGTGRA-3′) was excised from the gel and purified with the EasyPure Quick Gel Extraction Kit (TransGen Biotech, China) according to the manufacturer’s instructions. The DNA fragment was cloned into the pEASY-Blunt Cloning vector (TransGen Biotech, China). Ligated plasmids were transformed into *Escherichia coli* Trans5α competent cells according to the manufacturer’s protocol (TransGen Biotech, China). The cloned fragment was sequenced by Sangon Biological Engineering Technology and Services, Ltd. (Beijing, China) using the M13F and M13R vector-specific primers. Based on the sequence of the specific fragment, the SCAR marker (L60F/L60R) was designed by Primer-BLAST (https://www.ncbi.nlm.nih.gov/tools/primer-blast/) and synthesized by Sangon Biological Engineering Technology and Services, Ltd. (Beijing, China). The amplification was carried out in a 25-μl reaction mixture, which included 1 μl of DNA template (100 ng), 1 μl of the SCAR primer L60F (10 μM), 1 μl of the SCAR primer L60R (10 μM), 12.5 μl of 2 × EasyTaq PCR SuperMix (+dye) (TransGen Biotech, China), and 9.5 μl of ddH_2_O. The PCR amplification program was as follows: an initial denaturation (3 min at 94 °C), followed by 30 amplification cycles (30 s at 94 °C, 30 s at 57 °C, 45 s at 72 °C) and a final extension (10 min at 72 °C). We obtained a specific 660-bp band after the amplified products were separated by electrophoresis on a 1.0% (w/v) agarose gel (containing ethidium bromide) at 180 V for 20 min in 0.5 × TBE buffer, visualized on a UV transilluminator, photographed using a gel imager (Bio-Rad, USA), and then sequenced by Sangon Biological Engineering Technology and Services, Ltd. (Beijing, China).

### Specificity of the SCAR marker

To determine the specificity of the SCAR marker, we selected DNA from the pathogens based on three factors, except for the genomic DNA of *T*. *laevis* (5 isolates). First, we selected DNA from species that shared high similarity with *T*. *laevis*, including genomic DNA from *T*. *controversa* (5 isolates) and *T*. *tritici* (4 isolates). Second, we selected DNA from pathogens that cause disease in leaves and tassels of wheat, such as *P*. *striiformis* f. sp. *tritici* (3 isolates), *P*. *triticina* (3 isolates), *B*. *graminis* (5 isolates) and *F*. *graminearum* (5 isolates). Last, we selected DNA from species related to pathogens that cause smut disease in cereal crops, including *U*. *maydis* (5 isolates), *U*. *horde* (5 isolates), and *U*. *nuda* (5 isolates); ddH_2_O was used as a control. The DNA concentration of all tested isolates were adjusted to 1 ng/μl, and the amplification reaction mixture, the PCR amplification program and the amplified products were separated and sequenced as described above.

### Sensitivity of the SCAR marker

By using serial dilutions (4 ng/μl, 2 ng/μl, 1 ng/μl, 0.4 ng/μl, 0.04 ng/μl, 4 pg/μl, 0.4 pg/μl) of genomic DNA from *T*. *laevis*, the detection limit of the SCAR marker was determined. The amplification reaction mixture for PCR, amplification program and electrophoresis parameters were the same as those previously described.

### Real-time PCR detection

The fluorescence-based dye SYBR Green I was used for the real-time PCR (RT-PCR) experiment. The primer pair (qL60F: 5′-CTCTTCACTCGCATAGTCACTTG-3′/qL60R: 5′-GATGTTGGGTGGGTTACTGC-3′) was derived from the SCAR and synthesized by Sangon Biological Engineering Technology and Services, Ltd. (Beijing, China) (Fig. 2). To develop a standard curve, serial ten-fold dilutions of plasmid DNA (1.37 × 10^7^–1.37 × 10^2^ copies/μl, 0.01 ng–10 fg) were used as templates, which were established as follows: the volume of the TA cloning reaction system was 10 μL, consisting of 5 μL of 2 × Solution Buffer (ComWin Biotech, Beijing, China), 4 μL of recovered DNA bands, and 1 μL of T-vector (ComWin Biotech, Beijing, China); ligation was performed at 22 °C for 4 hours. TA cloning of the recovered PCR products was carried out using DH5α competent cells (ComWin Biotech, Beijing, China). Colonies were picked and dissolved in 10 μL of sterile water, and 1 μL of the above solution was used as a template for colony PCR. The colony PCR products were sequenced by Sangon Biological Engineering Technology and Services Co., Ltd. (Shanghai, China). The EasyPure^®^ Plasmid Mini Prep Kit (TransGen Biotech, Beijing, China) was used to extract plasmid DNA to be used as a standard for absolute quantification. For this purpose, the plasmid DNA standard was serially diluted 10-fold, and 2 μL of each dilution was used as a template to generate a standard curve. Then, RT-PCR was performed in a 20-μl reaction mixture containing 10 μl of 2 × PCR buffer (SYBR Premix Ex Taq II (RNaseH Plus), ROXplus), 0.5 μl of qL60F (10 μM), 0.5 μl of qL60R (10 μM), 7 μl of ddH_2_O and 2 μl of plasmid DNA sample. After the whole reaction system was mixed well and centrifuged, aliquots were loaded onto a 96-well PCR plate (0030128605, Eppendorf, Germany), and then three parallel RT-PCRs were designed for every sample. We also used 2 μL of nuclease-free water to replace the DNA template (2 μL) as a control on each plate. The ABI 7500 thermal cycler was used with the following reaction program: predenaturation at 95 °C for 30 s, followed by 40 cycles of denaturation at 95 °C for 5 s and annealing at 60 °C for 40 s. In addition, to generate melt curves, the following program was used: 95 °C for 1 min, 65 °C for 1 min, and a temperature rise from 65 °C to 95 °C at 0.5 °C/5 s. The fluorescent signal was measured at the annealing step and the extension step (60 °C for 40 s) of each cycle.

## Supplementary information

Submit
