# Supplementary material for: Development of ISSR-derived SCAR Marker and SYBR Green I Real-time PCR Method for Detection of Teliospores of Tilletia laevis Kühn
Source: Sci Rep. 2019 Nov 27;9:17651. doi: 10.1038/s41598-019-54163-5 (PMC6881473; doi:10.1038/s41598-019-54163-5)
Supplement: Supplementary file 1 — Submit [file 41598_2019_54163_MOESM1_ESM.pdf]

Development of ISSR-derived SCAR Marker and SYBR Green I Real-time PCR Method for Detection of Teliospores of *Tilletia laevis* Kühn

Zhaoqun Yao[1,2], Dandan Qin[1], Delai Chen[1,3], Changzhong Liu[3], Wanquan Chen[1*], Taiguo Liu[1], Bo Liu[1], Li Gao[1*]

[1]State Key Laboratory for Biology of Plant Disease and Insect Pests, Institute of Plant Protection, Chinese Academy of Agricultural Sciences, 100193, Beijing, China

[2]Key Laboratory at Universities of Xinjiang Uygur Autonomous Region for Oasis Agricultural Pest Management and Plant Protection Resource Utilization, Shihezi University, 832003, Shihezi, China

[3]College of Plant Protection, Gansu Agricultural University, 730070, Lanzhou, China

*e-mail: Li Gao (xiaogaosx@hotmail.com) Wanquan Chen (wqchen@ippcaas.cn)

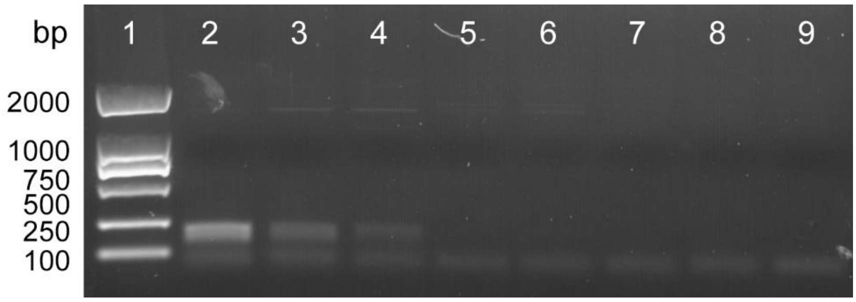

Figure S1. Sensitivity of the SC286-1/SC286-2 primers with different amounts of DNA templates. Lane 1: DL2000 DNA ladder, lane 2: 4 ng/µl, lane 3: 2 ng/µl, lane 4: 1 ng/µl, lane 5: 0.4 ng/µl, lane 6: 0.04 ng/µl, lane 7: 4 pg/µl, lane 8: 0.4 pg/µl, lane 9: ddH_2O

Table S1. Contrast of sensitivity and specificity between Real Time PCR and SCAR PCR

Fungi (Isolates Number)	DNA amounts (per µl)	SCAR PCR	Real Time PCR
Tilletia laevis (5)	0.4 ng	Yes	Yes
	10 fg	No	Yes
Tilletia controversa (5)	1 ng	No	-
Tilletia tritici (4)	1 ng	No	-
Puccinia striiformis f. sp. *tritici* (3)	1 ng	No	-
Puccinia triticina (3)	1 ng	No	-
Blumeria graminis (5)	1 ng	No	-
Fusarium graminearum (5)	1 ng	No	-
Ustilago maydis (5)	1 ng	No	-
Ustilago horde (5)	1 ng	No	-
Ustilago nuda (5)	1 ng	No	-

Note: “Yes” means can be detected, “No” means can not be detected, “-” means not available.
